# Control of microglial dynamics by the Arp2/3 complex and the autism- and schizophrenia-associated protein CYFIP1

**DOI:** 10.1073/pnas.2532488123

**Published:** 2026-03-12

**Authors:** James Scott-Solache, Jiaxin Pei, James Drew, Guillermo López-Doménech, Renaud B. Jolivet, Manuela Nieto-Rostro, Elizabeth C. Davenport, I. Lorena Arancibia-Cárcamo, David Attwell, Josef T. Kittler

**Affiliations:** ^a^Department of Neuroscience, Physiology and Pharmacology, University College London, London WC1E 6BT, United Kingdom; ^b^Maastricht Centre for Systems Biology and Bioinformatics, Faculty of Science and Engineering, Maastricht University, Maastricht 6229 EN, Netherlands; ^c^United Kingdom Dementia Research Institute at University College London, Institute of Neurology, Faculty of Brain Sciences, University College London, London WC1E 6BT, United Kingdom; ^d^The Cellular Phase of Alzheimer’s Disease Laboratory, The Francis Crick Institute, London NW1 1AT, United Kingdom

**Keywords:** 15q11.2, neuroinflammation, WRC, phagocytosis, glia

## Abstract

Microglia use a highly complex and dynamic network of branched processes to sense and respond to their surroundings. Despite emerging evidence that microglial motility plays important roles in brain development, neurodegeneration, and neuropsychiatric disease, little is known about the intracellular machinery orchestrating microglial process dynamics. Here, we identify roles for regulators of the actin cytoskeleton in controlling microglial behavior. We show that the actin branching Arp2/3 complex is critical for maintaining microglial morphology and is required for surveillance but not chemotactic motility. Neuropsychiatric disease-associated CYFIP1, a core component of the WAVE regulatory complex linking upstream signaling pathways to activation of the Arp2/3 complex, is highly expressed in microglia but has an unknown function. We report that conditional deletion of *Cyfip1* in mouse microglia reduces their morphological complexity and surveillance of the brain parenchyma, with no effect on chemotaxis. Deletion of *Cyfip1* also increased microglial CD68 positive lysosome volume and engulfment of presynapses. Thus, actin remodeling by CYFIP1 and the Arp2/3 complex controls microglial dynamics and shifts microglia away from a homeostatic state with potential implications for neuropsychiatric disease.

## Significance

Microglia, the resident immune cells of the brain, continuously survey their environment using a highly dynamic network of branched processes. Altered microglial morphology and behavior are increasingly linked to neurodevelopmental and neuropsychiatric disorders, yet the cytoskeletal mechanisms that generate and maintain ramified microglial structure remain unclear. We identify a crucial role for actin cytoskeleton remodeling via the Arp2/3 complex and the schizophrenia-associated regulator CYFIP1 in sustaining complex microglial morphology and effective surveillance of the brain. Cyfip1 deletion also increases synaptic cargo within microglial lysosomes, indicating a shift toward heightened phagocytic processing. These findings reveal cell-autonomous roles for CYFIP1 and Arp2/3-driven actin remodeling that may underlie microglial contributions to neuropsychiatric disease.

Microglia are the resident immune cells of the brain, performing innate immune functions during infection or damage. Imaging studies have shown that homeostatic microglia in the healthy brain are extremely motile, constantly surveying their surroundings using a complex network of branched processes (1–3). Subsequent research has revealed that surveying microglia interact with neurons to shape key aspects of brain development, including axon growth, synapse formation, and pruning (4–9). In parallel, clinical evidence suggests a pathological role for microglia in neurodegenerative and neuropsychiatric disorders, in which neuronal connectivity is often disrupted (10–14). How microglia generate ramified, dynamic protrusions and the implications of this for pathology remain poorly understood.

Cytoplasmic FMRP-interacting protein 1 (*CYFIP1*) is a gene with clinical associations with schizophrenia, ASDs, epilepsy, ADHD, and neurodevelopmental delay (15). In humans, *CYFIP1* resides in the q11.2 region of chromosome 15 that is vulnerable to microdeletions and duplications which are correlated with changes in protein expression (15–20). Further, associations between schizophrenia and mutations in *CYFIP1* altering mRNA levels have also been reported (21–24). Understanding the functions of CYFIP1 that could underlie these clinical associations is an active area of research.

A key function for CYFIP1 is in remodeling of the actin cytoskeleton. CYFIP proteins (of which there are two mammalian paralogs, CYFIP1 and CYFIP2) are constitutive members of the WAVE regulatory complex (WRC), a key regulator of the ubiquitous actin branching complex, Arp2/3 (25). The Arp2/3 complex, an actin nucleation factor, generates new filamentous actin branches from existing F-actin and is required for lamellipodial cell motility and invadopodia formation (26, 27). CYFIP proteins have a specific function in the WRC in coupling activation of the complex to upstream signaling from the small Rho GTPase Rac1 (28, 29). Importantly, the Rac1-CYFIP1-ARP2/3 pathway has well established roles in supporting neuronal growth and connectivity, with functional relevance to disease etiology. CYFIP1 deletion or haploinsufficiency in mice leads to defects in neuronal morphology, plasticity, and connectivity (30–34). These defects are coupled with perturbed actin remodeling dynamics and mirror clinical pathology. Alongside its role in actin remodeling, CYFIP1 acts as a cap-dependent translation repressor through its interactions with eIF4E and FMRP (36), and has been shown to regulate mTOR signaling in neurons in both mice (37) and flies (38).

In contrast to the wealth of literature on neuronal functions for CYFIP1, far less is known about its roles in other cell types in the brain. Interestingly, transcriptomic data from both human and murine tissues show that *CYFIP1* is widely expressed in glia, particularly microglia (39–41). Although microglia are highly branched cells and the actin cytoskeleton is required for microglial motility, specific functions for the actin remodeling machinery in microglia remain largely unknown. Thus, the fact that *CYFIP1* is a neuropsychiatric disease-associated risk gene could reflect its functions in actin remodeling in microglia (11, 42), which likely contribute to regulation of neuronal synaptic connectivity.

Here we show that maintenance of microglial morphology is critically dependent on sustained Arp2/3 complex activity. Inhibition of the Arp2/3 complex leads to a selective impairment in surveillance while ATP/ADP-driven chemotaxis remains intact. Using an inducible conditional *Cyfip1* knockout mouse, we show that deletion of microglial CYFIP1 recapitulates the effects of Arp2/3 complex inhibition on morphological complexity and impaired surveillance motility, with no effect on chemotaxis. We further show that this deletion leads to increased microglial lysosomal volume, with more presynaptic Bassoon present within these enlarged lysosomes. These findings uncover roles for CYFIP1 in mediating microglial dynamics and synapse phagocytosis, offering a mechanistic basis for the clinical associations of *CYFIP1*.

## Results

**Fig. 1.**
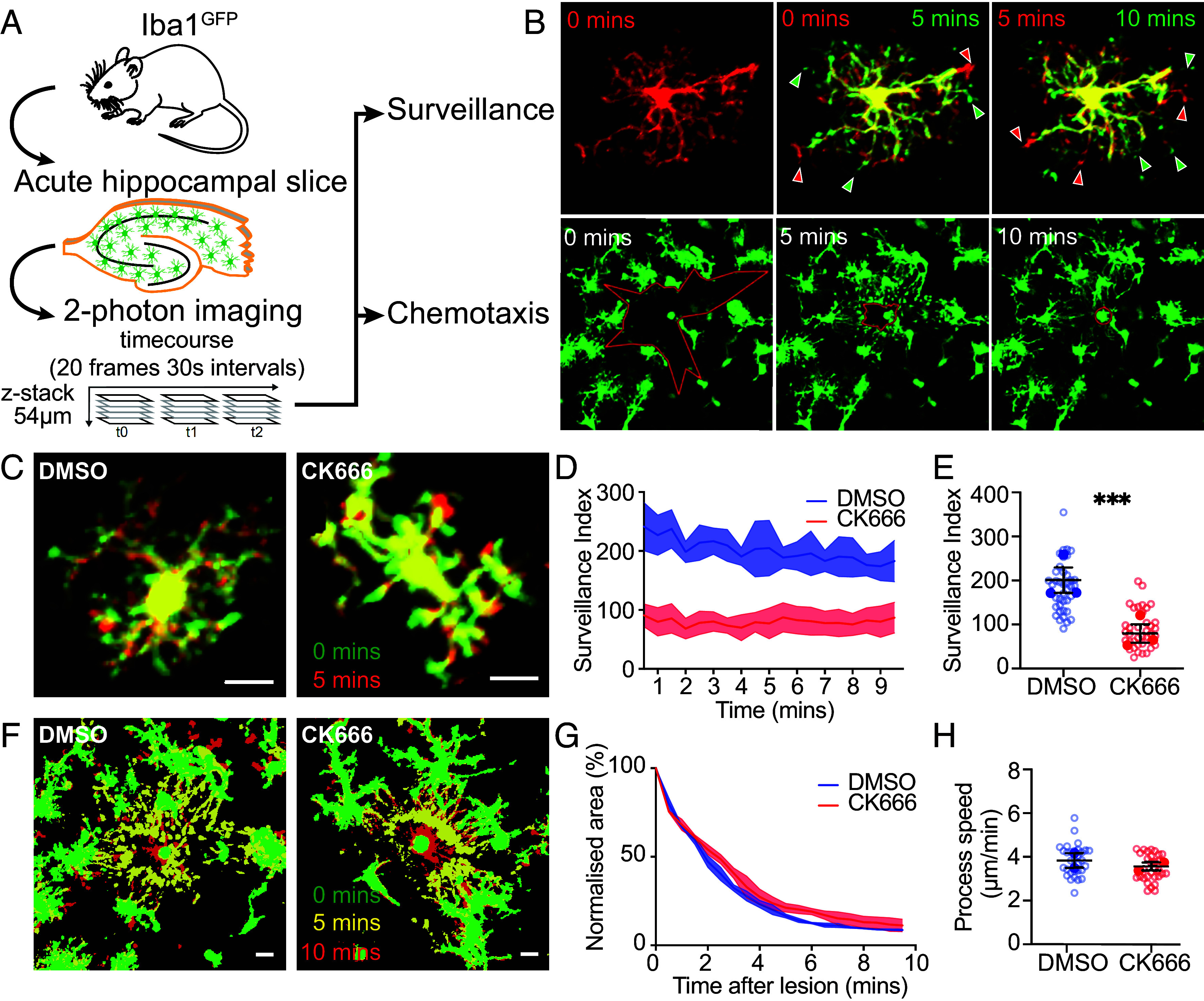
Inhibition of the Arp2/3 complex leads to defects in surveillance of brain parenchyma. (*A*) Schematic showing paradigm for 2-photon live imaging of surveillance and chemotaxis in acute hippocampal brain slices from Iba1^GFP^ mice. (*B*, *Top*) Example images depicting microglial surveillance analysis, demonstrating process movement over 5 min. (*Bottom*) Example images depicting microglial chemotaxis analysis, demonstrating process convergence to a site of laser damage after 5 and 10 min. (*C*) Superimposed timelapse images of representative cells from DMSO or CK666 treated slices, showing extensions (red), retractions (green), and stable regions (yellow) over 5 min (Scale bar: 10 μm.). (*D*) Quantification of surveillance from 10 min long, 30 s frame movies. (*E*) Surveillance index of CK666-treated cells decreased compared to controls (DMSO: 200.8 ± 28.85 pixels per 30 s, CK666: 79.66 ± 20.96 pixels per 30 s; *P* = 0.001; N = 4 animals, 38 to 39 cells). (*F*) Superimposed timelapse showing process chemotaxis toward circular lesion in both DMSO and CK666 treated slices (Scale bar: 10 μm.) (*G*) Quantification of process convergence (N = 2 animals, 6 lesions). (*H*) Single process tracking of CK666-treated processes (DMSO: 3.83 ± 0.34 μm/min^−1^, CK666: 3.56 ± 0.18 μm/min^−1^; *P* = 0.097; N = 2 animals, 31 cells). Data are presented as mean ± SEM, with per-brain slice averages shown as filled symbols and individual cell measurements as hollow circles. Linear Mixed Effects Model with slice as a source of variance, ns=nonsignificant *P < 0.05, **P < 0.01, ***P < 0.005, ****P < 0.001.

To capture microglial morphology and dynamics in situ, we performed 2-photon imaging of acute hippocampal brain slices from mice expressing eGFP under the microglia-specific Iba1 promoter (Fig. 1*A*) (43). Two forms of motility exhibited by microglia in vivo were assessed based on published protocols, namely spontaneous surveillance of surrounding parenchyma (“surveillance”) and chemotaxis of microglial processes toward damaged cells (“chemotaxis”) (44, 45). Briefly, random surveillance of the parenchyma by microglial processes was measured as the number of image pixels newly surveyed or retracted from, in the 30 s between frames (44), termed surveillance index (Fig. 1 *B*, *Upper*). Chemotactic response was quantified as the rate of process convergence toward a laser-induced lesion (Fig. 1 *B*, *Lower*). The requirement of actin polymerization for microglial motility was confirmed using cytochalasin D (10 μM), which essentially abolished both surveillance (*SI Appendix*, Fig. S1 *A*–*C*, *P* < 0.005) and chemotaxis (*SI Appendix*, Fig. S1 *F* and *G*, *P* < 0.01). In contrast, depolymerization of microtubules with vinblastine (10 μM) did not affect microglial surveillance over this timeframe (*SI Appendix*, Fig. S1 *D* and *E*).

The Arp2/3 complex plays a key role in driving filamentous actin branching. As little is known about the importance of Arp2/3 activity to microglial motility in situ, we began by treating microglia with the Arp2/3 complex inhibitor CK666 for 30 min prior to and during imaging (46). Analysis of surveillance revealed a >50% reduction in pixels surveyed by Arp2/3 complex-inhibited cells compared to vehicle (DMSO) treatment, for which surveillance remained approximately constant over the imaging period (Fig. 1 *C*–*E*, *P* < 0.005). Surprisingly, the chemotactic response to a laser-induced lesion remained robust under Arp2/3 complex inhibition (Fig. 1 *F*–*H*). The overall convergence of processes toward the lesion site was unchanged (Fig. 1*G* and *SI Appendix*, Fig. S1*I*), with no difference in the speed of individual processes observed (Fig. 1*H*).

**Fig. 2.**
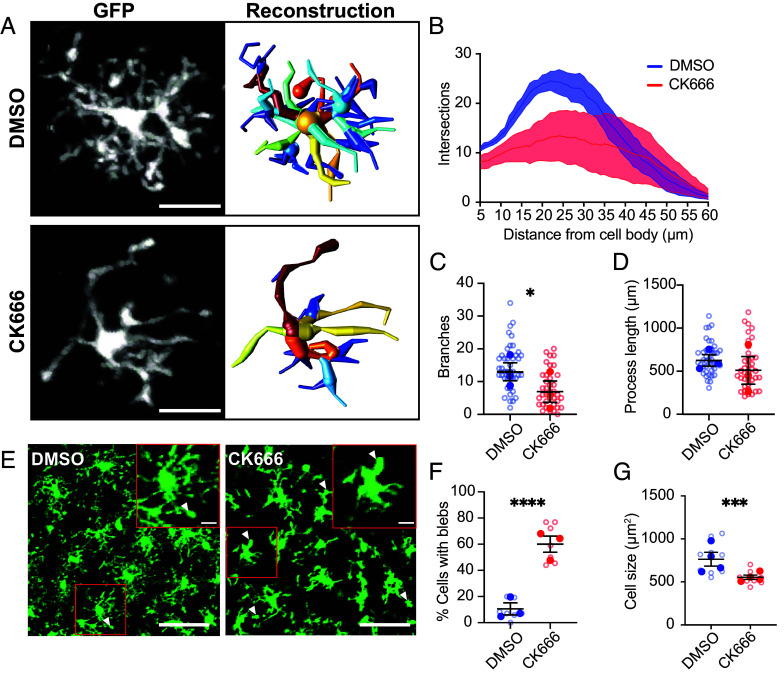
Arp2/3 complex activity is required to maintain ramification of microglia. (*A*) Representative cells from slices treated with DMSO (*Top*) or 200 μM CK666 (*Bottom*), showing loss of higher-order processors. (*Left*) Maximum intensity projection of GFP fluorescence. (*Right*) 3D reconstruction of cell morphology with color mapping to distinguish separate processes. (Scale bar: 10 μm.) (*B*) Sholl analysis of intersections from 3D reconstructions (Area under the curve, DMSO: 771.72 ± 72.04, CK666: 502.70 ± 196.79; *P* = 0.068; N = 3 animals, 38 to 42 cells). (*C*) Decrease in number of branch points (DMSO: 12.96 ± 2.748, CK666: 6.95 ± 3.25; *P* = 0.018; N = 3 animals, 38 to 42 cells). (*D*) Total process length (DMSO: 624.90 ± 66.77 μm, CK666: 509.90 ± 159.70 μm; *P* = 0.31; N = 3 animals, 38 to 42 cells). (*E*) CK666-treated microglia often had enlarged cellular protrusions. (*Left*) Field of view of CK666-treated slice highlighting “blebs” (white arrows). (Scale bars. Large FOV: 25 μm; Zoom: 5 μm.) (*F*) Quantification of proportion of microglia with blebs (DMSO: 10.50 ± 4.61 %, CK666: 60.04 ± 6.24 %; *P* < 0.0001; N = 3 animals, 7 FOVs). (*G*) Quantification of the area occupied by microglia in maximum intensity projected images from the first frame of surveillance analysis videos (DMSO: 765.40 ± 80.53 μm^2^, CK666: 554.40 ± 25.75 μm^2^; *P* = 0.009; N = 4 animals, 8 to 10 FOVs). Data are presented as mean ± SEM, with per-brain slice averages shown as filled symbols and individual cell measurements or slice as hollow circles. Linear Mixed Effects Model with slice as a source of variance, ns=nonsignificant *P < 0.05, **P < 0.01, ***P < 0.005, ****P < 0.001.

We observed that Arp2/3 complex-inhibited microglia displayed a notably different morphology to control cells and hypothesized that this could underlie the reduced surveillance phenotype. Specifically, CK666-treated cells showed a dramatic reduction in the complexity of microglial branching, characterized by retraction of secondary and tertiary process branches (Fig. 2*A*). 3D morphological reconstructions showed a reduction in the number of branch points in CK666-treated microglia compared to DMSO-treated (Fig. 2*C*, *P* < 0.05). There was a nonsignificant decrease in process arbor complexity in CK666-treated microglia by Sholl analysis (Fig. 2*B*, *P* = 0.068). Total process length was unaffected by CK666 (Fig. 2*D*). Primary processes often appeared swollen, with aberrant membrane blebbing observed at process tips (Fig. 2*E*). When quantified, approximately 60% of CK666-treated cells showed such blebbing, a sixfold increase over control (Fig. 2*F*, *P* < 0.001). Despite this blebbing, deramification of the CK666-treated microglia resulted in these cells occupying a smaller area within each field of view (Fig. 2*G*, *P* < 0.005). These data suggest that Arp2/3 complex activity is required for the normal ramified morphology of microglia, and effective surveillance, but is dispensable for chemotaxis. A similar mechanistic difference between surveillance and chemotaxis has been demonstrated when inhibiting the main microglial K^+^ channel THIK-1 (45).

**Fig. 3.**
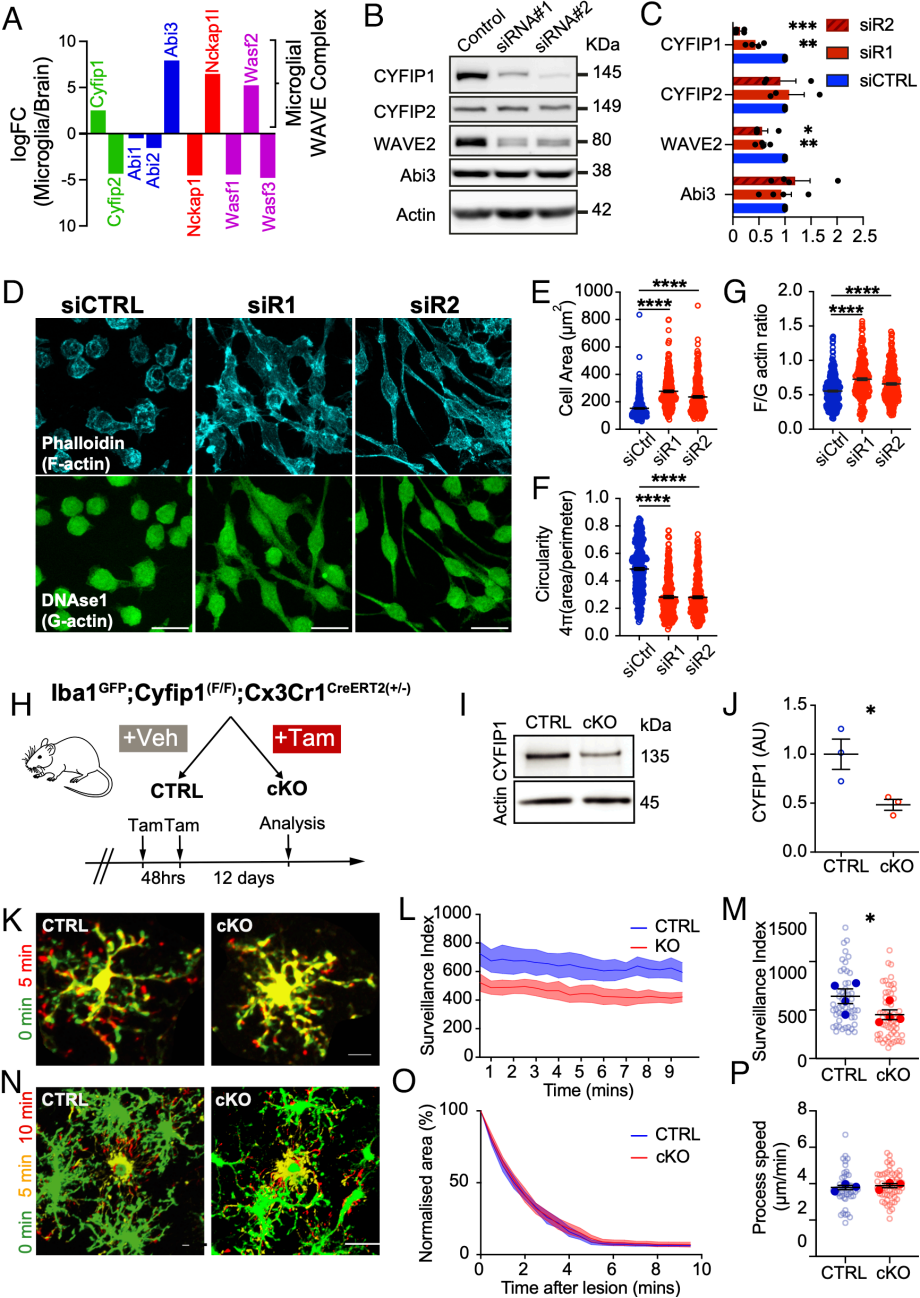
Knockout of Cyfip1 disrupts actin remodeling and surveillance activity. (*A*) mRNA expression of WRC components, showing log(fold change) (logFC) of FPKM values from microglia and whole brain samples. Positive logFC values indicates enrichment in microglia. Adapted from Zhang et al. (40). See also *SI Appendix*, Fig. S2*A*. (*B*) Example Western blots showing expression levels of other wave regulatory complex members, CYFIP2, WAVE2, and Abi3, following knockdown of CYFIP1 using two different siRNAs. (*C*) Quantification of protein expression of CYFIP1 (siCTRL: 1 ± 0, siR1: 0.43 ± 0.01, siR2: 0.14 ± 0.005, vs. siR1 *P* < 0.01, vs. siR2 *P* < 0.001, four individual experiments), CYFIP2 (siCTRL: 1 ± 0, siR1: 1.07 ± 0.30, siR2: 0.92 ± 0.29, vs. siR1 *P* = 0.731, vs. siR2 *P* = 0.52, three individual experiments), WAVE2 (siCTRL: 1 ± 0, siR1: 0.57 ± 0.06, siR2: 0.57 ± 0.10, vs. siR1 *P* < 0.05, vs. siR2 *P* < 0.01, four individual experiments), and Abi3 (siCTRL: 1 ± 0, siR1 0.92 ± 0.20, siR2: 1.20 ± 0.28, vs. siR1 *P* = 0.73, vs. siR2 *P* = 0.52, four individual experiments) following knockdown of CYFIP1. (*D*) ICC for phalloidin and DNAse1 in control or *Cyfip1-*targeting siRNA-treated BV2 cells. (*E*) Quantification of cell area (siCTRL: 152.67 ± 4.05 μm^2^, siR1: 277.20 ± 7.90 μm^2^, siR2: 236.30 ± 7.69 μm^2^, siCTRL vs. siR1 *P* < 0.0001, siCTRL vs. siR2 *P* < 0.0001, one-way ANOVA with Bonferroni post hoc, n = 246 to 357 cells from four experiments). Data are presented as mean ± SEM. (*F*) Quantification of cell circularity measured as 4π(area/perimeter^2^) (siCTRL: 0.49 ± 0.01, siR1: 0.28 ± 0.01, siR2: 0.28 ± 0.01, siCTRL vs. siR1 *P* < 0.0001, siCTRL vs. siR2 *P* < 0.0001, one-way ANOVA with Bonferroni post hoc, n = 246 to 357 cells from four experiments). (*G*) Quantification of the ratio of F- to G-actin (siCTRL: 0.56 ± 0.01, siR1: 0.80 ± 0.03, siR2: 0.67 ± 0.017, siCTRL vs. siR1 *P* < 0.0001, siCTRL vs. siR2 *P* < 0.0001, one-way ANOVA with Bonferroni post hoc, n = 246 to 357 cells from four experiments). Data are presented as mean ± SEM, with individual cell measurements shown as filled symbols. (*H*) Schematic showing generation of conditional *Cyfip1* deletion in microglia using the CreERT2driven from the microglia-specific CX3CR1 promoter. Cre was activated through treatment of experimental mice with tamoxifen. See also *SI Appendix*, Fig. S3*A*. (*I*) Western blot for CYFIP1 in an enriched microglial extract from adult mouse brain. (*J*) Quantification of CYFIP1 levels in enriched microglial extracts from Control and cKO mice (control: 1 ± 0.15, cKO: 0.48 ± 0.06, *P* < 0.05, Student’s *t* test, n = 3 lysates preparations). (*K*) Superimposed timelapse images of microglia from control or cKO brain slices, showing extensions (red), retractions (green), and stable regions (yellow) over 5 min. (Scale bar: 10 μm.) (*L*) Quantification of surveillance from 10 min long, 30 s frame movies. (*M*) Surveillance index of cKO microglia decreased compared to controls (control: 642.40 ± 75.95 pixels per 30 s, cKO: 452.20 ± 50.13 pixels per 30 s; *P* = 0.023; N = 4 animals, 52 to 56 cells). (*N*–*P*) Chemotaxis is normal in cKO microglia. (*N*) Superimposed timelapse showing process chemotaxis in control and cKO slices. (Scale bar: 20 μm.) (*O*) No change in process convergence. (*P*) Single process tracking shows normal extension speed (control: 3.79 ± 0.12 μm/min; cKO: 3.89 ± 0.12 μm/min; *P* = 0.475; N = 3 animals, 48 to 59 cells). Data are presented as mean ± SEM, with per-brain slice averages shown as filled symbols and individual cell measurements as hollow circles. Individual *t* tests (*C* and *J*), One-Way ANOVA with Bonferroni post hoc correction (*E*–*G*) or Linear Mixed Effects Model (*M* and *P*), ns=nonsignificant *P < 0.05, **P < 0.01, ***P < 0.005, ****P < 0.001.

CYFIP proteins are the molecular link between upstream signaling and WRC-dependent Arp2/3 complex activity. An analysis of microglial expression of paralogs of WRC components from published RNA sequencing revealed a canonical “microglial WRC” composed of *Cyfip1*, *Abi3*, *Nap1l* (*Nckap1l*), and *Wasf2* (*WAVE2*) (Fig. 3*A*) (40). Notably, *Cyfip1* was expressed ~2.6 times higher in microglia compared to other brain cells, whereas *Cyfip2* expression is virtually absent in microglia in both humans and mice (*SI Appendix*, Fig. S2*A*). To demonstrate a link between the loss of CYFIP1 and perturbed actin remodeling, we utilized two in vitro models of CYFIP1 loss. Knockdown (KD) of *Cyfip1* in the BV2 microglial cell line using two different siRNAs resulted in a ~40% loss of WAVE2 expression (Fig. 3 *B* and *C*, siRNA #1 *P* < 0.01, siRNA #2 *P* < 0.05), WAVE2 being the subunit that contains the VCA domain that is required to activate Arp2/3 complex activity (47), without affecting protein expression of the AD risk gene, ABI3. *Cyfip1-*KD BV2 cells were larger (Fig. 3 *D* and *E*, *P* < 0.0001), had lost their characteristic circular morphology in favor of a bipolar morphology (Fig. 3*F*, *P* < 0.0001), and had an increased F-actin to G-actin ratio (Fig. 3*G*, *P* < 0.0001). Conditional knockout of CYFIP1 in mouse embryonic fibroblasts (MEFs) generated from *Cyfip1* floxed mice (*SI Appendix*, Fig. S2*B*) also resulted in an increased F-actin to G-actin ratio (*SI Appendix*, Fig. S2*D*, *P* < 0.0001), as well as a significant reduction in the number of cells with lamellipodia (*SI Appendix*, Fig. S2 *F* and *G*, *P* < 0.001), indicative of disrupted Arp2/3 complex activity (48)–and fewer actin stress fibers per cell (*SI Appendix*, Fig. S2 *H* and *I*, *P* < 0.01). Given these data, we hypothesized that CYFIP1 could play a role in regulating microglial dynamics upstream of the Arp2/3 complex.

To explicitly investigate this, we generated a microglia-specific *Cyfip1* conditional knockout (cKO) mouse model. A floxed *Cyfip1* mouse was crossed with a line expressing an inducible CreERT2 fusion protein driven by the microglia-specific *Cx3cr1* promoter and the *Iba1*^GFP^ reporter line used previously to visualize microglia in situ. Induction of Cyfip1 recombination was initiated by two consecutive oral 4 mg tamoxifen doses between P28 and P30 (Fig. 3*H* and *SI Appendix*, Fig. S3*A*) (31, 35, 49). Recombination of the *Cyfip1* cassette was confirmed by PCR of brain tissue from control or *Cyfip1-*cKO animals (*SI Appendix*, Fig. S3*B*) and enriched microglial protein lysates from cKO brains showed a significant decrease in CYFIP1 expression of approximately 50% over control (Fig. 3 *I* and *J*, *P* < 0.05), showing that Cre induction in this model leads to specific loss of microglial CYFIP1 in vivo.

**Fig. 4.**
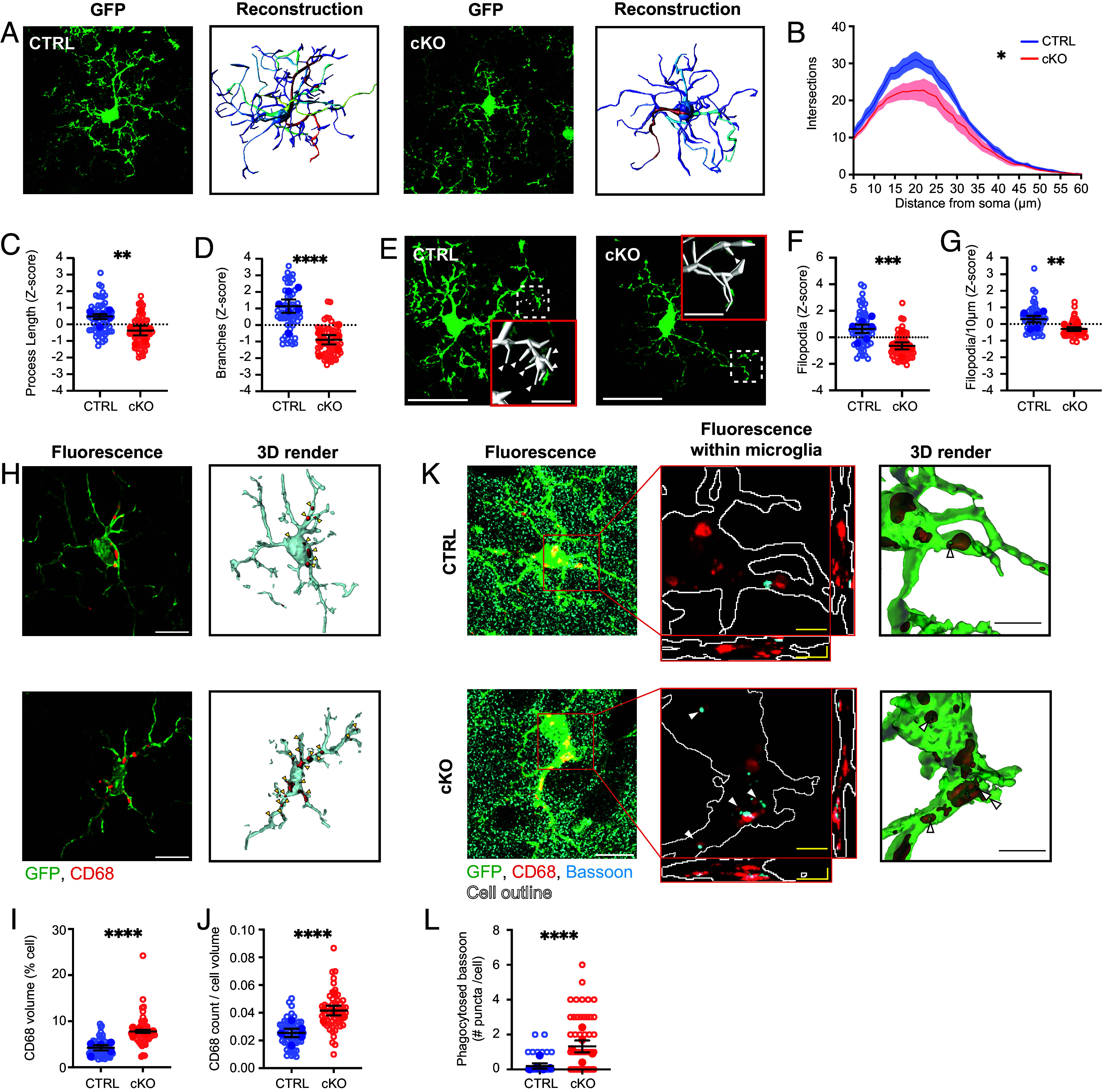
Decreased morphological complexity and synapse engulfment of *Cyfip1-*cKO microglia. (*A*) Representative projections of GFP IHC (*Left*) and 3D reconstructions (*Right*) of microglia taken from fixed slices of control and *Cyfip1-*cKO brains, showing reduced complexity of arborizations. (*B*) Sholl analysis of reconstructions reveal a decrease in the number of intersections across the arbor (Area under the curve, *P* = 0.031; N = 6 animals, 62 to 63 cells), (*C*) reduced total process length (Z-score; *P* = 0.009; control 0.478 ± 0.147, cKO: −0.370 ± 0.291) (*D*) and reduced number of branch points (Z-score; *P* < 0.001; control: 1.144 ± 0.405, cKO: −0.887 ± 0.283). (*E*) Microglial filopodia decorate processes (white arrows, *Top Right*) and can be automatically reconstructed by Vaa3D (*Bottom Right*). (*F*) Quantification of the number of filopodia shows reductions in cKO cells both when measured per cell (Z-score; *P* < 0.001; control: 0.640 ± 0.318, cKO: −0.640 ± 0.262, n = 6 animals, 63 cells) (*G*) or per 10 μm of process (Z-score; *P* = 0.0018; control: 0.603 ± 0.378, cKO: −0.603 ± 0.213). Filopodia were defined as process tips with length <5 μm. (*H* and *I*) CD68 staining in cKO microglia in the somatosensory cortex. (*H*) Example images of immunofluorescence (*Left*) and 3D volume renders (*Right*) of CD68 staining in single microglia. Arrowheads indicate individual lysosomes. (Scale bar, 10 μm.) (*I*) Quantification reveals increase in CD68 burden in cKO cells of the somatosensory cortex as a percentage of total cell volume (*P* < 0.001; control: 4.25 ± 0.58 %, cKO: 7.49 ± 0.29 %; N = 5 animals, 54 to 56 cells). (*J*) and increased number of discrete lysosomes within microglia relative to cell size (*P* < 0.001; control: 0.025 ± 0.003, cKO: 0.042 ± 0.004; N = 5 animals, 54 to 56 cells). (*K* and *L*) Bassoon presence within microglial lysosomes in the somatosensory cortex. (*K*) Example images of immunofluorescence (*Left*; Scale bar, 10 μm), zoomed in views of Bassoon within the microglia (*Middle*; Scale bar, 2 μm), and 3D volume renders (*Right*; Scale bar, 2 μm) of Bassoon within CD68+ lysosomes of single microglia. Arrowheads indicate Bassoon puncta within lysosomes (*L*). Quantification reveals increase in number of Bassoon puncta within lysosomes in cKO cells of the prefrontal cortex (*P* < 0.001; control: 0.19 ± 0.15 puncta/cell, cKO: 1.32 ± 0.35; *P* = 0.018; n = 5 animals, 55 to 56 cells). Data are presented as mean ± SEM, with per-animal averages shown as filled symbols and individual cell measurements as hollow circles. Linear Mixed Effects Model, ns = nonsignificant *P < 0.05, **P < 0.01, ***P < 0.005, ****P < 0.001.

We first investigated microglial motility in control and *Cyfip1-*cKO animals using the two assays of surveillance and chemotaxis described previously. Quantifying surveillance of cKO microglia compared to control showed a 33% reduction in surveillance index (Fig. 3 *K*–*M*, *P* < 0.05). Interestingly, loss of CYFIP1 had no effect on the chemotactic responses to laser-induced lesion (Fig. 3 *N*–*P*), as was observed with Arp2/3 complex inhibition. We hypothesized that impaired surveillance in the *Cyfip1-*cKO microglia was due to morphological defects in these cells. To test this, control and cKO brains were fixed, and 100 μm sections were GFP signal amplified or immunostained with Iba1+P2RY12 (for *Iba1*^GFP^ negative animals) to enable 3D reconstructions of individual hippocampal microglia (Fig. 4*A*) (50). Sholl analysis revealed a decrease in the complexity of microglial processes that appeared consistent across the whole arbor (Fig. 4*B*, *P* < 0.05). Supporting this, total process length and number of branch points were both significantly reduced in the *Cyfip1-*cKO (Fig. 4 *C* and *D*, *P* < 0.01 and *P* < 0.001 respectively, *SI Appendix*, Fig. S3 *D* and *E*). Microglial processes are decorated with many highly dynamic filopodia that support surveillance of the brain parenchyma (51). These structures were clearly visible in our fixed tissue (Fig. 4*E*) but were not included in our initial reconstructions. To specifically investigate filopodia, a lower minimum threshold for process length (1 μm instead of 5 μm) was used for reconstructions, and the number of tips within the 1 to 5 μm length range was defined to represent the number of filopodia. Interestingly, the number of filopodia per cell was decreased by 46% in cKO microglia (Fig. 4*F*, *P* < 0.01, *SI Appendix*, Fig. S3*F*). This effect remained even when normalized to the total process length (Fig. 4*G*, *P* < 0.01; *SI Appendix*, Fig. S3*G*), suggesting that this is not simply a consequence of the reduced arbor complexity seen in Fig. 4*C*. Unlike morphology, there was no effect of *Cyfip1*-cKO on microglial density compared to controls (*SI Appendix*, Fig. S3 *H* and *I*).

Finally, we investigated whether loss of CYFIP1 had any broader impact on microglial state or function. CYFIP1 has been associated with regulating the function of mTOR complex 1 (mTORC1) in neurons (37, 38). mTORC1 is crucial for controlling many aspects of microglial activity including inflammation (52), metabolism (53), and phagocytosis (54, 55). Staining of fixed sections for phosphorylated ribosomal protein S6 (pS6), a readout of mTORC1 activity, found cortical *Cyfip1*-cKO microglia had an increased pS6 intensity compared to controls (*SI Appendix*, Fig. S4*A*, *P* < 0.05). This effect was also observed in *Cyfip1*-KO MEFs (*SI Appendix*, Fig. S2 *C* and *E*, *P* < 0.0001). This conserved increased pS6 level in *Cyfip1*-KO cells suggests CYFIP1 plays a role in restraining mTORC1 activity in microglia.

Microglia are the professional phagocytes of the brain, engulfing unwanted material from their environment and degrading them within their lysosomes (56). To see whether loss of CYFIP1 alters lysosome presence in microglia, brain sections were stained for CD68 and the lysosomal number and size within microglia were quantified, based on a published protocol (57). *Cyfip1*-KO microglia had an increased volume and number of CD68 positive structures as a percentage of the total cell (Fig. 4 *H*–*J*, *P* < 0.001 and *P* < 0.001 respectively). As microglia are known to be involved in the phagocytic removal of synapses, sections were stained for the presynaptic marker, Bassoon, and the number of Bassoon puncta within microglial lysosomes was quantified. As expected based on the enlarged lysosomes, *Cyfip1*-cKO microglia had significantly more Bassoon within their lysosomes (Fig. 4 *K*–*L*, *P* < 0.001). Together with the morphological and surveillance defects described above, these data highlight the importance of CYFIP1 for normal microglial physiology and interaction with their environment.

## Discussion

Microglial interactions within the brain during both health and disease are critically dependent on their shape and motility. Here, we report that microglial ramification is dependent on both sustained activity of the Arp2/3 complex, and expression of the neuropsychiatric disease-associated Arp2/3 complex regulator CYFIP1. Targeting either of these components of the actin branching machinery led to a specific defect in surveillance of the brain parenchyma, whereas chemotaxis remained robust.

The ramified shape of surveying microglia forms dynamically from the extension and retraction of processes (58, 59). Our observation that reduced microglial ramification and dysmorphic process blebbing was observed after only 30 min of Arp2/3 complex inhibition implies that the complex is tonically active in surveying microglia. The higher-order processes, which are more motile and mainly responsible for surveillance over the timeframe that we imaged for, were preferentially affected by CK666 treatment, leading to severely impaired surveillance of the brain parenchyma. The bleb-like processes seen during Arp2/3 complex inhibition are reminiscent of alternative actin-based protrusions used by amoeboid cells to migrate through 3D environments. Indeed, blebbing is stimulated by inhibition of the Arp2/3 complex in other cell types (60–62).

Arp2/3 complex inhibition specifically impacts surveillance over chemotaxis. The Arp2/3 complex has been suggested to regulate the localization of focal adhesions required to interact with the extracellular matrix (ECM)(48, 63). The specific impact of Arp2/3 complex inhibition on surveillance but not chemotaxis may be due to processes extending toward the laser lesion in this assay relying primarily on actin polymerization rather than interactions with the extracellular matrix (64). Arp2/3 complex inhibition also specifically impairs surveillance but not chemotaxis in other types of macrophage (64). Furthermore, our observations fit with other studies in microglia showing divergence in the signaling pathways underlying surveillance and chemotaxis, the potassium channel THIK-1 controls surveillance but is dispensable for chemotaxis, conversely the ADP receptor P2Y12 is only required for chemotaxis (45, 51, 65, 66). Given that both forms of motility remain critically dependent on actin, as seen with cytochalasin D treatment, it is remarkable that the divergence of signaling underlying these behaviors extends down to the machinery regulating actin filament formation.

CYFIP1 has well-established roles in Arp2/3 complex activation, via its presence in the WAVE regulatory complex (26, 67). Here, we used a conditional knockout model to show that microglia-specific deletion of CYFIP1 leads to impaired surveillance of the brain parenchyma and reduced morphological complexity of these cells, phenotypes similar to those seen during inhibition of the Arp2/3 complex. Thus, we hypothesize that CYFIP1 deletion leads to disruption of the WRC and Arp2/3 complex activation.

As well as reducing microglial surveillance activity and morphological complexity, *Cyfip1* deletion also increased lysosomal volume and synaptic content within lysosomes. As microglia from patients with schizophrenia display elevated expression of phagocytic genes (including CD68) (68) and phagocytosis of synapses (12), the increased phagocytosis of synapses by microglia due to CYFIP1 deletion may constitute a potential mechanism underlying the association between *CYFIP1* variants and disease risk. Interestingly, *CYFIP1* deletion reduces phagocytosis of synaptosomes in vitro in human induced pluripotent stem cell (iPSC)-derived microglia-like cells (69). This raises the possibility that knockout of *Cyfip1* might disrupt the phagolysosome in microglia in our study, resulting in increased synaptic material within the lysosome through reduced degradation rather than an increase in phagocytic uptake. Importantly, the Arp2/3 complex plays a role in lysosome integrity and function (70). Genetic deletion of *Abi3*, another member of the WRC, also increases CD68 volume in microglia (71). This effect of increased lysosomal cargo due to reduced degradation has been seen in microglia lacking other genes including progranulin (72) and TFEB (73).

Accompanying the increased synaptic material within enlarged lysosomes in *Cyfip1*-cKO microglia is an elevated mTORC1 activity. Knockout of the mTOR repressor, *Tsc1*, in microglia similarly results in elevated S6 phosphorylation, increased lysosomal volume, as well as microglial phagocytosis, while pharmacological inhibition or genetic deletion of mTOR reverses these phenotypes (54, 55, 74). It is unclear how loss of CYFIP1 is increasing mTORC1 activity in our models as previous work has shown that CYFIP1 expression was positively correlated with mTORC1 activity in neurons (37, 38)–the opposite to the effect demonstrated here in microglia. Whether loss of Cyfip1 is increasing synaptic phagocytosis by increasing mTORC1 activity or if increased mTORC1 activity is a product of disrupted actin remodeling and independent of phagocytosis is currently unclear and warrants further investigation.

A key advantage of our cKO model is the ability to discern microglial-specific effects of CYFIP1 deletion. Most in vivo studies of CYFIP1 have relied on heterozygous models, where CYFIP1 expression is altered in all cells, making inferring the cell autonomy of phenotypes extremely challenging (33, 75, 76). This is highlighted by two studies on rodent CYFIP1 haploinsufficient models that both reported myelin thinning but drew different conclusions as to whether altered neuronal activity or oligodendrocyte function underpinned these effects (30, 77).

Microglia are in active and intimate association with neurons during key stages in development, which helps to shape neuronal connectivity (6, 9, 78, 79). These interactions occur within the surveillance field of a microglial cell and could well be affected by alterations in the cell’s ramification and motility. Interestingly, other WRC components expressed in microglia are increasingly implicated in neurological disorders with microglial pathologies. *WAVE2* was found to be differentially expressed in microglia from ASD individuals (13) and *ABI3*, exclusively expressed by microglia in the brain, is a risk gene for Alzheimer’s disease (80, 81). Thus, we provide direct support for disease-relevant functions for CYFIP1 and the WAVE regulatory complex in microglia, and illustrate the need to investigate the cell autonomous roles of these genes to properly understand their contribution to pathology.

## Materials and Methods

### Experimental Model and Subject Details.

#### Animals.

Animals of either sex were used for all experiments. All procedures for the care and treatment of animals were in accordance with the Animals (Scientific Procedures) Act 1986 and had full Home Office ethical approval. All animals were maintained under controlled conditions (temperature 20 ± 2 °C; 12 h light–dark cycle). Food and water were provided ad libitum. Animals were group housed in conventional cages and had not been subject to previous procedures.

#### Mouse line generation and inducible Cre recombinase activation.

The *Cyfip1* f/f mouse line (MDCK; EPD0555_2_B11; Allele: Cyfip1^tm2a(EUCOMM)Wtsi^) was generated previously (31). *Cyfip1* conditional knockout animals were generated by crossing *Cyfip1^F^*^/F^ animals with the Cx3cr1^tm2.1(cre/ERT2)^ line. Microglia were visualized for two-photon live imaging experiments by crossing conditional knockout animals with an Iba1^GFP^ line. All surveillance imaging was done using Iba1^GFP^ mice (which avoid the loss of function inherent in using even heterozygote CX3CR1-GFP knock-in mice). Confocal imaging of fixed immunostained slices used both Iba1^GFP^ expressing and nonexpressing mice as indicated in the figure legend.

To activate Cre in the Cx3cr1^(Cre/ERT2)^ line, the estrogen receptor agonist tamoxifen was administered to adult mice at P28 and again at P30 via oral gavage. Tamoxifen was dissolved in 40 mg/mL solution of 90% corn oil (Kolliphor EL, Sigma):10% ethanol by sonication and two doses of 4 mg/100 μL were administered, 48 h apart. Tamoxifen solution was made fresh for each dosing regimen and kept at 4 °C between doses. Control animals were administered 100% corn oil. Experiments were carried out, 2 wk after the second administration, at P44. This is an age at which microglia are in a stable homeostatic state (78, 82, 83).

#### Isolation of enriched microglia fraction.

To generate protein lysates of enriched adult microglia from transgenic mice, a Percoll-based isolation method was used as described previously (84). Briefly, three mice per experimental condition were transcardially perfused with ice-cold PBS, brains extracted, and finely minced using a flat scalpel blade in 3 mL HBSS. Tissue was enzymatically digested in a dispase-papain-DNAase solution and triturated into a single cell suspension. The suspension was then loaded into a 70%:37%:30% Percoll/HBSS gradient. After centrifugation without brakes, myelin debris migrated to the 30%:37% interface and microglial cells were enriched in the 70%:37% interface. The microglia fraction was extracted, washed, and lysed in sample buffer.

#### Acute slice preparation.

To prepare acute hippocampal slices, male and female mice aged postnatal day 28 to 34 were used for experiments involving drug treatments and at postnatal day 44 for experiments involving tamoxifen or corn oil treated animals. Immediately after decapitation, the brain was removed and kept in ice-cold dissecting solution (in mM: 87 NaCl, 25 NaHCO_3_, 10 glucose, 75 sucrose, 2.5 KCl, 1.25 NaH_2_PO_4_, 0.5 CaCl_2_, and 7 MgCl_2_) saturated with 95%O_2_/5% CO_2_. Transverse hippocampal slices (300 μm) were obtained using a vibratome (Leica, VT–1200S). Slices were transferred to HEPES-buffered ACSF (in mM: 140 NaCl, 10 glucose, 10 HEPES, 2.5 KCl, 1 MgCl_2_, 2 CaCl_2_, 1 NaH_2_PO_4_, saturated with 100% O_2_ (pH 7.4) at 22 °C, and left to recover for 30 min prior to use.

### Methods Details.

#### Antibodies.

For immunocytochemistry experiments the following primary antibodies or dyes were used: Phalloidin-ATTO647 (65906-Sigma Aldrich 1:2,000), DNAseI-488 (D12371-ThermoFisher 1:500), anti-GFP (GFP-1020-Aves lab 1:1,000), anti-Iba1 (234 004-Synaptic Systems 1:1,000), Anti-P2Y12R (AS-550434A AnaSpec, 1:2,000) anti-CD68 (MCA1957GA-BioRad 1:500), anti-Bassoon (SAP7F407 – Novus Bioloigicals 1:1,000), and anti-Phospho-S6 (D68F8-CellSignaling 1:1,000). For western blot experiments the following antibodies were used: anti-CYFIP1 (07-531-Upstate 1:1,000), anti-CYFIP2 (ab79716-Abcam 1:500), anti-actin (A2066-Sigma Aldrich 1:5,000), anti-WAVE2 (sc33548-SantaCruz 1:500), and anti-Abi3 (sc376982-SantaCruz 1:500),

#### Two-photon imaging and drug treatments.

Acute slices (see Acute slice preparation above) of Iba1^GFP^ animals were imaged in continually perfused HEPES-buffered aCSF using a two-photon microscopy system (Zeiss LSM 7 MP system, Mai Tai SpectraPhysics lasers). Slices were imaged between 30 min to 4 h after slicing. The hippocampus was found using brightfield illumination and regions containing well-labelled, unactivated microglia between 50 to 150 μm from the slice surface were used for timelapse recordings. For surveillance measurements, z-stacks of 52 μm regions (26 optical sections, 2 μm interval, 512 × 512 pixels) were taken every 30 s for 10 min, except for cytochalasin D treatment which was taken every 60 s for 10 min. *Cyfip1-*cKO experiments were imaged at 2× zoom, whereas drug treatment experiments were imaged at 1.5× zoom, as the stronger treatment effects permitted imaging of more cells within a wider field of view at lower resolution. For chemotaxis measurements, a circular ROI of 15 μm diameter was selected in an area surrounded by labelled microglia. High-powered laser excitation (90% power) within the ROI was used to create a lesion in the slice, using the bleaching plugin in Zen 2010 (12 iterations, 2 speed). Immediately after lesion, 3D movies were taken using the same imaging parameters as for surveillance.

For DMSO (1%) and CK666 (200 μM) treatments, acute slices were incubated for 30 min prior to imaging in a low-volume incubation chamber with drug solutions in HEPES-buffered ACSF. Slices were then imaged for a maximum of 30 min using a reperfusion system that enabled recycling of drug-containing imaging solution. For cytochalasin D (10 μm) and Vinblastine (10 μm) treatments, acute slices were incubated for 10 min in a low-volume incubation chamber with drug solutions in HEPES-buffered ACSF prior to imaging in normal ASCF.

#### Transcardial perfusion fixation.

Brains were fixed by transcardial perfusion (TCP) of 4% paraformaldehyde (PFA) (4% PFA, PBS). Animals underwent terminal anesthesia via intraperitoneal injection of 1 μL/g pentobarbital solution. When fully anesthetized, 10 to 15 mL of ice-cold 4% PFA was perfused into the left ventricle using a peristaltic pump, the animal decapitated, brain removed, and placed in ice-cold 4% PFA overnight. Certain protocols required TCP of PBS to remove all blood from the brain. In these cases, the protocol above was used except that ice-cold PBS was perfused.

#### Immunohistochemistry.

Immunohistochemistry of *Cyfip1*-cKO animals was carried out on perfusion-fixed brains from litter matched pairs. Resected brains were transferred to PBS and 20 or 100 μm slices made using a vibratome (Leica Microsystems, Heerbrugg, Switzerland).

Free floating sections were washed in PBS before permeabilization in blocking solution (10% horse serum, 0.5% BSA, 0.2% Triton X-100, PBS) for 2 to 4 h and incubation with primary antibody diluted in block solution overnight at 4 °C. For mouse primary antibodies, slices were first incubated overnight at 4 °C with mouse Fab fragment (1:50 with blocking solution). Slices were washed 4 to 5× in PBST for 2 h then incubated for 3 to 4 h with secondary antibody at RT. Slices were then washed 4 to 5× in PBS for 2 h and mounted onto glass slides using Mowiol mounting medium. In IBA1^GFP(−/−)^ mice, microglia were costained with P2Y12R and IBA1 primary antibody both detected with Alexa Fluor 488-conjugated secondary antibodies, enabling a comprehensive visualization of microglia morphology. For Sca*l*eS4 cleared brain slices, stained slices were incubated in Sca*l*eS4 reagent (85) for 3 h at 37 °C before being mounted on glass slides using Sca*l*eS4 reagent as mounting medium. For cryoslices, brains were dehydrated with 30% sucrose/PBS solution and frozen at −80 °C before embedding in OCT (Optimal Control Temperature). 30 μm sections were made using a Cryostat (Bright Instruments, Luton, UK) and stored at −20 °C as free-floating slices in cryoprotect solution (30% ethylene glycol, 30% glycerol, 40% PBS). For antigen retrieval, slices were incubated in sodium citrate solution at 80°C for 40 min and then washed 3× in PBS prior to blocking.

#### Generation of mouse embryonic fibroblasts (MEFs).

A *Cyfip1* conditional knockout MEF line was generated by crossing *Cyfip1^F^*^/F^ animals with the Rosa26-Cre-ERT2 line (86). Generation and immortalization of the MEF line was performed essentially as described (87). Briefly, E10.5 embryos were harvested on ice-cold dissection buffer. The heads and viscera were removed from the embryos, and the remaining tissue was cut in pieces and incubated with 30 μL 0.125% trypsin for 10 min at 37 °C. The trypsin was removed and tissue was washed twice with dissection buffer and gently triturated by pipetting repeatedly (5 to 10 strokes) in DMEM complete medium. Cells were then plated on 6 cm dishes and were passaged repeatedly until they gained immortal characteristics. After 8 to 10 passages at low density, the cultures presented a homogeneous cell population and started to grow steadily. Transformed cell lines were then genotyped to confirm genetic background. All experiments were carried out after at least eight passages from the immortalization process.

Cre-induced recombination was promoted by treating the culture with 4-hydroxytamoxifen (OHT) at 2 mM for six consecutive days. Experiments were performed the day after the end of the treatment.

#### CYFIP1 silencing experiments.

Silencing of *Cyfip1* was performed on the microglial derived BV2 cell line. We obtained three predesigned Silencer™ Select siRNAs to target *Cyfip1* of which two were used in all experiments based on silencing power (siRNA IDs: s73706 and s73707) and one negative control (catalog numbers: 4390771 and 4390846 from Thermofisher). We transfected the siRNAs in BV2 cells using the Lipofectamine RNAiMax (Thermofisher) following the manufacturer’s instructions. Experiments were performed 72 h after siRNA transfection.

### Quantification and Statistical Analysis.

#### Analysis of live imaging data.

For chemotaxis analysis, maximum intensity projections (MIPs) of raw images were preprocessed using a custom ImageJ macro that involved: XY registration, background subtraction (50 px), maximum filtering (2 px). As part of z-registration, single z-planes at the extremes of the stack were sometimes removed. Preprocessed MIPs were manually thresholded and the resulting movies processed using a custom previously published MATLAB script (45). Briefly, after the user manually selected the lesion region, the algorithm divides the surrounding area into concentric circles with radii at 2 μm intervals, and then segments these circles into 32 radial sectors, thus creating 32 patches between every two consecutive concentric circles. Then, for each frame, starting from the center, the algorithm searches in every radial sector for the first patch containing >10 positive pixels (labeled microglia). The outputs of the algorithm are, for each frame, i) the distance to the microglial process front in each sector and ii) the surface area contained within the converging microglial process front.

For surveillance analysis, regions containing single cells were cropped from raw images. Crops were preprocessed using a separate ImageJ macro that involved: XYZ registration, background subtraction (50 px), maximum filtering (2 px), and 3D-bleach correction. Finally, GFP signal from surrounding cells was removed using the Clear function in ImageJ to isolate motility of a single cell. MIPs from these processed images were binary thresholded and processed in a MATLAB script published in Madry et al. (45). For each movie, starting with the second frame, we subtracted from each binarized frame F_t_ the preceding frame F_t−1_ and created two binarized movies, PE consisting of only the pixels containing process extensions (F_t_ − F_t−1_ > 0) and PR consisting of only the pixels containing process retractions (F_t_ − F_t−1_ < 0). In both PE and PR, all other pixels are set to 0. The surveillance index at each timepoint was defined as the sum of PE and PR, and an average of surveillance index across the movie used to generate a single value for each cell.

#### Quantifications of microglial morphology.

Microglial morphology in *Cyfip1*-cKO animals was assessed using perfusion-fixed brains from litter matched pairs. Thick sections underwent IHC (see Transcardial perfusion fixation and immunohistochemistry) to amplify the GFP signal or immunostain for P2Y12R and IBA1. High-resolution z-stacks of single microglia were taken using a Zeiss LSM700 upright confocal microscope using a 63X oil objective (NA: 1.4) (70 to 100 μm z-stacks; xy resolution: 0.10 μm; z step: 0.32 μm, 1,024 × 1,024 px). Input z-stack images were preprocessed using a custom ImageJ macro to subtract background (50 px [0.5 μm] rolling ball average), median filter (2 px [0.2 μm] radius), and improve contrast (manual adjustment). Semiautomated 3D reconstructions were produced using the “APP2” tracing plugin in Vaa3D software (http://home.penglab.com/proj/vaa3d), and manually checked with any clear abnormalities corrected. Morphological parameters of reconstructions were extracted using custom MATLAB code (available at https://github.com/AttwellLab). For morphology data in drug treatment experiments, the first frame of each live imaging movie was used as the raw data for generating reconstructions described above.

#### Microglial lysosome and presynapse phagocytosis analysis.

Analysis of CD68 positive lysosomes as well as intralysosomal Bassooon within individual microglia were performed based on a published protocol by Schafer et al. (57). For quantifying lysosomal presence within microglia, thick (100 μm) brain sections were immunostained for CD68 and either GFP (For Iba1-eGFP+ animals) or Iba1+P2RY12. Individual somatosensory cortical microglia were confocal imaged (70 to 100 μm z-stacks, xy resolution: 0.10 μm; z step: 0.50 μm, 1,024 × 1,024 px). Particle analysis of CD68 volume render was used to calculate total volume and counts of lysosomal structures, and both were normalized to the total volume of the cell render where required. For quantification of phagocytosed Bassoon, 20 μm free-floating sections were antibody stained for CD68, Iba1, and Bassoon and individual microglia within the somatosensory cortex were confocal imaged (10 μm z-stacks, xy resolution: 0.10 μm; z step: 0.50 μm, 512 × 512 px). Images were preprocessed in ImageJ to remove background and smoothen by median filtering (radius = 1 pixel) before creating surface renders of the target microglia, lysosomes within the microglia, and Bassoon within lysosomes using Imaris software. The number of discrete Bassoon puncta within microglial lysosomes was quantified.

#### Statistical analysis.

All statistical analysis was carried out using GraphPad Prism (GraphPad Software, CA, USA) or the Python statsmodels package. For all experiments involving animals, linear mixed-effects models (LMEMs) were used to account for the nonindependence of cells nested within animals while testing for treatment or genotype effects (88). In *Cyfip1-*cKO experiments, the model included genotype as a fixed effect and animal identity as a random effect:

*model = statsmodels.formula.api.mixedlm(“Readout ~ Group”, dataframe, groups=dataframe[“Animal”])*.

For experiments involving drug treatments (e.g., CK666), where drug treatments were applied to individual brain slices and not directly to the animal, the brain slice from which the measured cell data comes from was included as a variance component (vc):

*model = statsmodels.formula.api.mixedlm(“Readout ~ Group”, dataframe, groups=dataframe[“Animal”], vc_formula={“Slice”: “0 + C(Animal_Slice)”})*.

For Sholl analyses, repeated measures across radii were summarized as a single area-under-the-curve (AUC) value per cell. These AUC values were then analyzed using LMEMs as above.

*Cyfip1-*cKO morphology data were acquired from both Iba1-GFP^+/−^ with GFP amplification and Iba1-GFP^−/−^ animals immunostained with Iba1 and P2RY12 in the same fluorescent channel, with data from three animals per genotype acquired for each method. To assess the impact of *Cyfip1-*cKO on microglial morphology independent of methodology, data were normalized by z-scoring all cells within each method. Specifically, for each method, raw values were transformed to z-scores by subtracting the group mean and dividing by the group SD: z = (x − μ)/σ. This procedure ensured comparability of morphology measurements across labeling methods, while preserving biological variability between genotypes. Z-scored values were then used as the input for LMEM analysis as described above.

Cell culture experiments (BV2 microglia and MEFs) were analyzed using classical parametric statistics (*t* tests or ANOVA) with Bonferroni post hoc corrections for multiple testing when appropriate.

Data are shown as mean ± SEM. Where relevant, each datapoint representing an animal is represented as a filled circle and individual cell measurements are shown as unfilled circles to depict data spread. Statistical tests, *P*-values, and number of biological replicates are given in the figure legends. N numbers refers to number of animals unless otherwise stated.

## Supplementary Material

Appendix 01 (PDF)

Dataset S01 (XLSX)

## Data Availability

Study data are included in the article and/or supporting information.
