# Supplementary material for: Control of microglial dynamics by the Arp2/3 complex and the autism- and schizophrenia-associated protein CYFIP1
Source: Proc Natl Acad Sci U S A. 2026 Mar 12;123(11):e2532488123. doi: 10.1073/pnas.2532488123 (PMC12993954; doi:10.1073/pnas.2532488123)
Supplement: Supplementary file 1 — Appendix 01 (PDF) [file pnas.2532488123.sapp.pdf]

PNAS

Supporting Information for

Control of microglial dynamics by the Arp2/3 complex and the autism- and schizophrenia-associated protein CYFIP1

James Scott-Solache[1,*], **Jiaxin Pei**[1,*], **James Drew**[1,2,*], **Guillermo López-Doménech**[1], **Renaud B. Jolivet**[1,3], **Manuela Nieto-Rostro**[1], **Elizabeth C. Davenport**[1,4], **I. Lorena Arancibia-Cárcamo**[1,5,6], **David Attwell**,[1, 2] and **Josef T. Kittler** [1,+]
[1] Department of Neuroscience, Physiology and Pharmacology, University College London, Gower Street, London WC1E 6BT, UK. [2] Present Address: Citeline, London, UK. [3] Maastricht Centre for Systems Biology and Bioinformatics (MaCSBio), Maastricht University, Paul-Henri Spaaklaan 1, 6229 EN Maastricht, Netherlands. [4]Present Address: Centre for Discovery Brain Sciences, University of Edinburgh, Edinburgh, UK. [5] UK Dementia Research Institute at UCL, University College London, London, UK. [6] The Francis Crick Institute, London, UK.

Correspondence: Josef T. Kittler
Email: j.kittler@uc.ac.uk

This PDF file includes:

Figures S1 to S4

Supplementary Figures

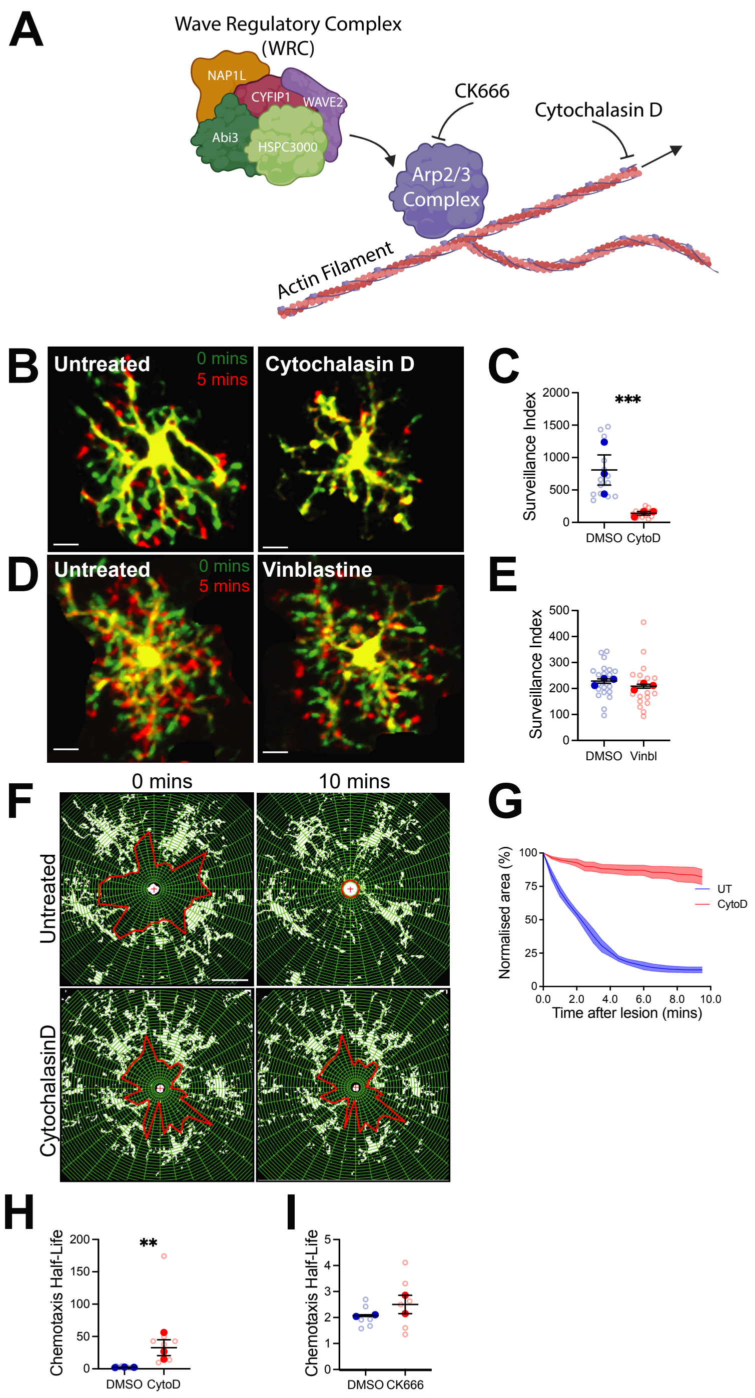

Figure S1. Microglial motility requires actin but not microtubule polymerisation.
(A) Schematic depiction of the Rac1-WRC-Arp2/3 actin remodelling and the drugs used in this study that disrupt this pathway. Arp2/3 is inhibited by CK666 while Cytochalasin D is an inhibitor of actin filament elongation by preventing actin polymerisation. (B-C) 10 μM Cytochalasin D

treatment abolishes microglial surveillance (B) Superimposed maximum intensity projection of representative cells at 0 and 5 minute timepoints (green = retraction; red = extension; yellow = stable). (C) Average of surveillance index over the course of each movie (p=0.0045; untreated: 808.40 ± 232.80 px per 30 sec, cytochalasin D: 139.60 ± 27.80 px per 30 sec; N=3 animals, 14-15 cells). (D-E) Depolymerisation of microtubule cytoskeleton with 10 µM vinblastine does not affect microglial surveillance. (D) Superimposed maximum intensity projection of representative cells at 0 and 5 minute timepoints (green = retraction; red = extension; yellow = stable). (E) Average of surveillance index over the course of each movie (p=0.489; untreated: 228.6 ± 8.47 px per 30 sec, vinblastine: 208.7 ± 7.482 px per 30 sec; N=3 animals 18-21 cells). (F) Binarised max projections of untreated and cytochalasin D microglia. Convergence of processes seen as a reduction of red polygon area that is unchanged in cytochalasin D condition. (G) Change in area (normalised to starting area) of polygon over time. (H) Average half-life of decay of the normalised area of polygon over time in CytochalasinD-treated brain slices (p=0.0088; untreated: 2.39 ± 0.228, CytochalasinD: 32.61 ± 12.26; N=3 animals; 8-9 lesions. (I) Average half-life of decay of the normalised area of polygon over time in CK666-treated brain slices (p=0.340; DMSO: 2.07 ± 0.033, CK666: 2.50 ± 0.352; N=2 animals, 6 lesions).
Linear Mixed Effects Model, ns=non-significant *$P < 0.05$, **$P < 0.01$, ***$P < 0.005$, ****$P < 0.001$

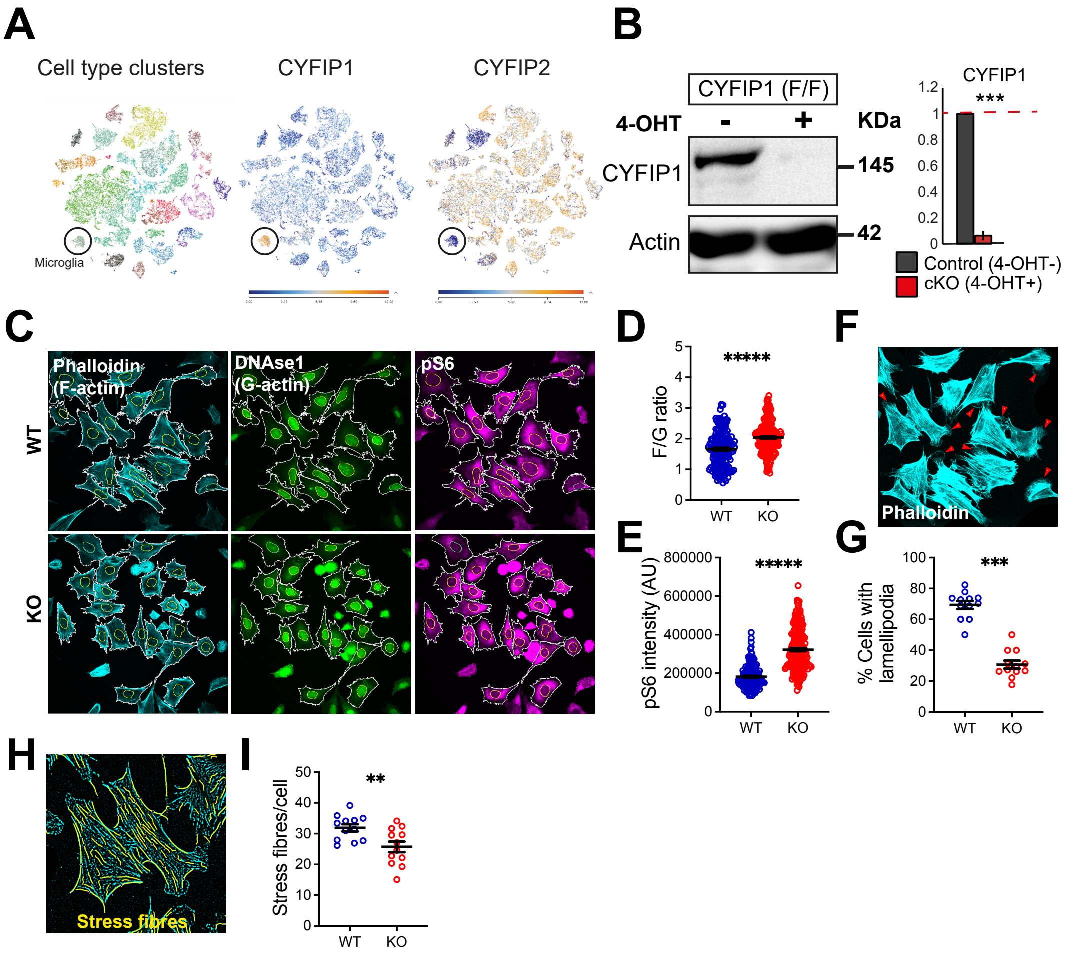

Figure S2. Knockout of Cyfip1 affects morphology of mouse embryonic fibroblasts (MEFs) (A) t-SNE plots of single cell sequencing data from human cortex from the Allan Brain Map by the Allan Institute (https://celltypes.brain-map.org/rnaseq/human/cortex, accessed 10.04.2020). Left: Colour mapping highlights clusters identifying specific cell populations, including clearly distinct microglia cluster (circle). Middle and right: CYFIP gene expression mapped onto cell clusters, showing that microglia are specifically enriched in CYFIP1 (middle) over CYFIP2 (right). Data from: Hodge, R.D., Bakken, T.E., et al. (2019). (B) Example Western blot and quantification of reduction of CFYIP1 in MEFs following 4-OHT treatment (p<0.0001; WT: 1 ± 0, KO: 0.061 ± 0.029; N=3 experiments). (C) ICC for phalloidin, DNAse1, and pS6 in WT and KO MEFs. (D) Quantification of the ratio of F- to G-actin (p<0.0001; WT: 1.66 ± 0.05, KO: 2.04 ± 0.04; N=163-198 cells from 3 experiments). (E) increased pS6 presence in cKO MEFs. pS6 integrated density is increased in cKO MEFs (p<0.0001; WT: 181.76 ± 4.73, KO: 322.33 ± 7.87; N=163-198 cells from 3 experiments). (F) ICC for phalloidin in WT MEFs, with red arrowheads highlighting lamellipodia. (G) Quantification of the proportion of cells with lamellipodia (p<0.0001; WT: 69.30 ± 0.2.55%, KO: 30.70 ± 2.55%; N=12 fields of view from 3 experiments) (H) Depiction of method for quantification of actin stress fibres in MEFs. (I) Quantification of the number of stress fibres per cell (p<0.01; WT: 31.91 ± 1.18, KO: 25.72 ± 1.71; N=12 fields of view from 3 experiments) T-tests, ns=non-significant *P < 0.05, **P < 0.01, ***P < 0.005, ****P < 0.001

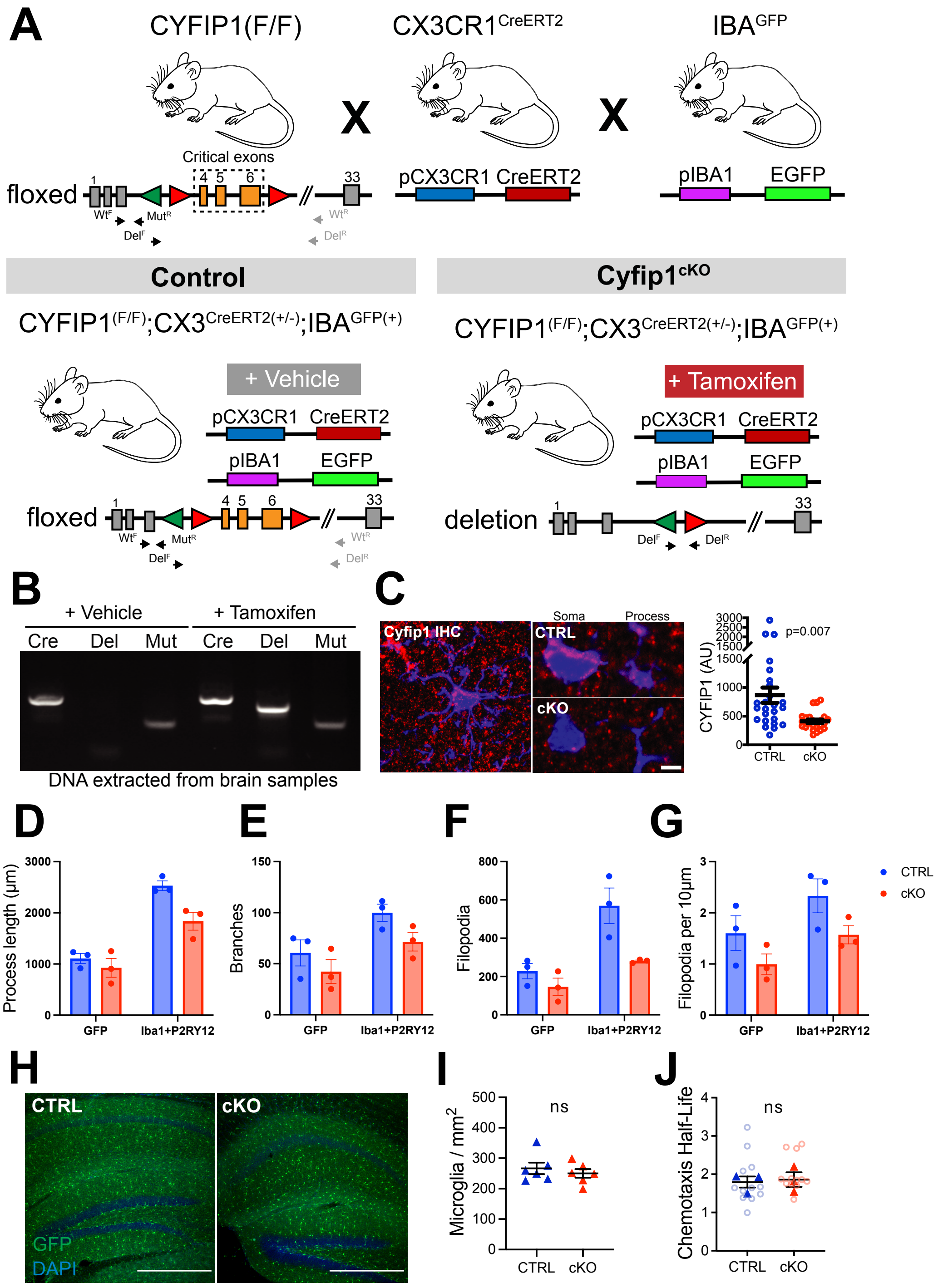

Figure S3. Generation of Cyfip1 conditional knockout mouse model.
(A) Schematic illustrating the genetic modifications used to generate the Cyfip1 conditional knockout (cKO) mouse model. A floxed Cyfip1 line was generated using a knockout-first strategy and bred to homozygosity in all parental mice. To generate knockout alleles, floxed Cyfip1 mice were crossed with a knock-in CX3Cr1CreERT2 line. CX3Cr1CreERT2 allele was kept heterozygous in all breeding and experimental animals. An IBAGFPline used previously was crossed in to fluorescently label microglia. Recombination of the floxed Cyfip1 locus excises

critical exons 4-6, leading to loss of functional Cyfip1. Nuclear translocation of Cre recombinase was activated by tamoxifen treatment and was inactive in vehicle treatment. Small arrowheads identify approximate sites for PCR primer sequences for wildtype (WtF/WtR), conditional (WtF/MutR) and the deletion (DelF/DelR) alleles of the Cyfip1 locus. Grey arrows denote no PCR product. (B) PCR confirmation of recombination of the floxed Cyfip1 cassette in control and Cyfip1 cKO mice. (C) IHC for Cyfip1 in IBA^{GFP} control and cKO mice. Left panel: max projections of Cyfip1 staining. Right panels: zooms of soma and processes show reduction of Cyfip1 puncta in cKO microglia. Scale bar: 10 μm (large), 2 μm (zoom). Quantification of Cyfip1 levels in the cell soma (control: 867.70 ± 132 Integrated density, cKO: 410.20 ± 30.85 Integrated Density; $p<0.05$, Mann-Whitney; n=25 cells from 3 animals). (D-G) Depiction of the effect of genotype and method on (D) average process length, (E) number of branch points, (F) number of filopodia, and number of filopodia per 10μm of cell process. See Figure 4C-D. (H) Immunohistochemistry of GFP+ microglia (green) and nuclei (DAPI; blue). Scale bar: 500μm. (I) Quantification of microglial density in the hippocampus CA1 as microglia per mm^2 ($p=0.684$; control: 266.60 ± 18.89, cKO 250.40 ± 14.19; N=6 animals). (J) Average half-life of decay of the normalised area of polygon over time in Cyfip1-cKO brain slices ($p=0.497$; control: 1.70 ± 0.16 cKO: 1.97 ± 0.20; N=3 animals, 12 lesions).

T-test (C) and Linear Mixed Effects Model (I&J), ns=non-significant *$P < 0.05$, **$P < 0.01$, ***$P < 0.005$, ****$P < 0.001$

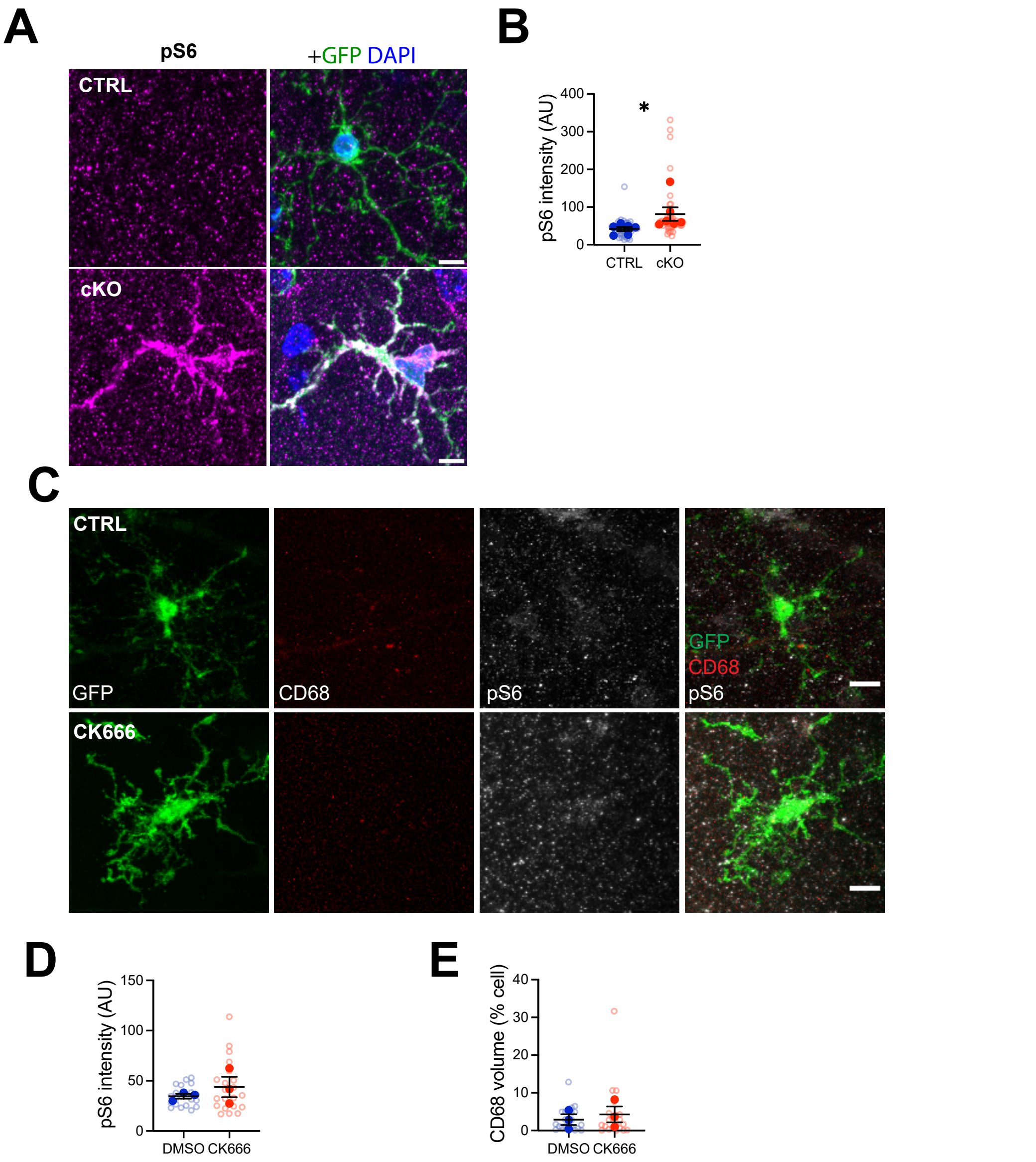

Figure S4. Effects of Cyfip1-cKO and CK666 on mTORC1 activity in microglia.
(A) pS6 staining in Control and cKO microglia. Example images of immunofluorescence of microglia (GFP) and pS6 in microglia. Scale bar: 5µm. (B) Quantification of increased pS6 integrated density in cKO microglia normalised to cell volume (p=0.043; control: 41.95 ± 5.39, cKO: 81.22 ± 17.91; N=6 animals, 51 cells. (C) IHC of microglia (GFP), CD68, and pS6 in control and CK666 treated acute brain slices. Scale bar: 5µm. (D) Quantification reveals no difference in pS6 integrated density normalised to cell volume (p=0.089; DMSO: 34.74 ± 2.40, CK666: 43.89 ± 10.17; N=3 animals, 21 cells) (E) nor CD68 volume (p=0.398; DMSO: 2.87 ± 1.43%, CK666: 4.29 ± 2.11%; N= 3 animals, 21 cells) between control and CK666 treated microglia.
Linear Mixed Effects Model with (D&E) and without (B) the slice as a source of variance, ns=non-significant *$P < 0.05$, **$P < 0.01$, ***$P < 0.005$, ****$P < 0.001$
